# Tuning 2D magnetism in Fe_3+X_GeTe_2_ films by element doping

**DOI:** 10.1093/nsr/nwab117

**Published:** 2021-07-02

**Authors:** Shanshan Liu, Zihan Li, Ke Yang, Enze Zhang, Awadhesh Narayan, Xiaoqian Zhang, Jiayi Zhu, Wenqing Liu, Zhiming Liao, Masaki Kudo, Takaaki Toriyama, Yunkun Yang, Qiang Li, Linfeng Ai, Ce Huang, Jiabao Sun, Xiaojiao Guo, Wenzhong Bao, Qingsong Deng, Yanhui Chen, Lifeng Yin, Jian Shen, Xiaodong Han, Syo Matsumura, Jin Zou, Yongbing Xu, Xiaodong Xu, Hua Wu, Faxian Xiu

**Affiliations:** State Key Laboratory of Surface Physics and Department of Physics, Fudan University, Shanghai 200433, China; Institute for Nanoelectronic Devices and Quantum Computing, Fudan University, Shanghai 200433, China; State Key Laboratory of Surface Physics and Department of Physics, Fudan University, Shanghai 200433, China; Institute for Nanoelectronic Devices and Quantum Computing, Fudan University, Shanghai 200433, China; College of Science, University of Shanghai for Science and Technology, Shanghai 200093, China; Laboratory for Computational Physical Sciences (MOE), Fudan University, Shanghai 200433, China; State Key Laboratory of Surface Physics and Department of Physics, Fudan University, Shanghai 200433, China; Institute for Nanoelectronic Devices and Quantum Computing, Fudan University, Shanghai 200433, China; Solid State and Structural Chemistry Unit, Indian Institute of Science, Bangalore 560012, India; School of Electronic Science and Engineering, Nanjing University, Nanjing 210093, China; Department of Physics, University of Washington, Seattle, WA 98195-1560, USA; Department of Electronic Engineering, Royal Holloway University of London, Egham TW20 0EX, UK; Materials Engineering, The University of Queensland, Brisbane QLD 4072, Australia; Beijing Key Laboratory of Microstructure and Property of Advanced Materials, Institute of Microstructure and Property of Advanced Materials, Beijing University of Technology, Beijing 100124, China; The Ultramicroscopy Research Center, Kyushu University, Fukuoka 819-0395, Japan; The Ultramicroscopy Research Center, Kyushu University, Fukuoka 819-0395, Japan; State Key Laboratory of Surface Physics and Department of Physics, Fudan University, Shanghai 200433, China; Institute for Nanoelectronic Devices and Quantum Computing, Fudan University, Shanghai 200433, China; State Key Laboratory of Surface Physics and Department of Physics, Fudan University, Shanghai 200433, China; Institute for Nanoelectronic Devices and Quantum Computing, Fudan University, Shanghai 200433, China; State Key Laboratory of Surface Physics and Department of Physics, Fudan University, Shanghai 200433, China; Institute for Nanoelectronic Devices and Quantum Computing, Fudan University, Shanghai 200433, China; State Key Laboratory of Surface Physics and Department of Physics, Fudan University, Shanghai 200433, China; Institute for Nanoelectronic Devices and Quantum Computing, Fudan University, Shanghai 200433, China; Department of Electronic Engineering, Royal Holloway University of London, Egham TW20 0EX, UK; State Key Laboratory of ASIC and System, School of Microelectronics, Fudan University, Shanghai 200433, China; State Key Laboratory of ASIC and System, School of Microelectronics, Fudan University, Shanghai 200433, China; Beijing Key Laboratory of Microstructure and Property of Advanced Materials, Institute of Microstructure and Property of Advanced Materials, Beijing University of Technology, Beijing 100124, China; Beijing Key Laboratory of Microstructure and Property of Advanced Materials, Institute of Microstructure and Property of Advanced Materials, Beijing University of Technology, Beijing 100124, China; State Key Laboratory of Surface Physics and Department of Physics, Fudan University, Shanghai 200433, China; Institute for Nanoelectronic Devices and Quantum Computing, Fudan University, Shanghai 200433, China; Collaborative Innovation Center of Advanced Microstructures, Nanjing 210093, China; State Key Laboratory of Surface Physics and Department of Physics, Fudan University, Shanghai 200433, China; Institute for Nanoelectronic Devices and Quantum Computing, Fudan University, Shanghai 200433, China; Collaborative Innovation Center of Advanced Microstructures, Nanjing 210093, China; Beijing Key Laboratory of Microstructure and Property of Advanced Materials, Institute of Microstructure and Property of Advanced Materials, Beijing University of Technology, Beijing 100124, China; The Ultramicroscopy Research Center, Kyushu University, Fukuoka 819-0395, Japan; Department of Applied Quantum Physics and Nuclear Engineering, Kyushu University, Fukuoka 819-0395, Japan; Materials Engineering, The University of Queensland, Brisbane QLD 4072, Australia; Centre for Microscopy and Microanalysis, The University of Queensland, Brisbane QLD 4072, Australia; School of Electronic Science and Engineering, Nanjing University, Nanjing 210093, China; Department of Physics, University of Washington, Seattle, WA 98195-1560, USA; State Key Laboratory of Surface Physics and Department of Physics, Fudan University, Shanghai 200433, China; Laboratory for Computational Physical Sciences (MOE), Fudan University, Shanghai 200433, China; Collaborative Innovation Center of Advanced Microstructures, Nanjing 210093, China; State Key Laboratory of Surface Physics and Department of Physics, Fudan University, Shanghai 200433, China; Institute for Nanoelectronic Devices and Quantum Computing, Fudan University, Shanghai 200433, China; Collaborative Innovation Center of Advanced Microstructures, Nanjing 210093, China; Shanghai Research Center for Quantum Sciences, Shanghai 201315, China

**Keywords:** 2D ferromagnetic material, Fe3+XGeTe2 film, element doping, above room temperature, *T*C tunability

## Abstract

Two-dimensional (2D) ferromagnetic materials have been discovered with tunable magnetism and orbital-driven nodal-line features. Controlling the 2D magnetism in exfoliated nanoflakes via electric/magnetic fields enables a boosted Curie temperature (*T*_C_) or phase transitions. One of the challenges, however, is the realization of high *T*_C_ 2D magnets that are tunable, robust and suitable for large scale fabrication. Here, we report molecular-beam epitaxy growth of wafer-scale Fe_3+X_GeTe_2_ films with *T*_C_ above room temperature. By controlling the Fe composition in Fe_3+X_GeTe_2_, a continuously modulated *T*_C_ in a broad range of 185–320 K has been achieved. This widely tunable *T*_C_ is attributed to the doped interlayer Fe that provides a 40% enhancement around the optimal composition X = 2. We further fabricated magnetic tunneling junction device arrays that exhibit clear tunneling signals. Our results show an effective and reliable approach, i.e. element doping, to producing robust and tunable ferromagnetism beyond room temperature in a large-scale 2D Fe_3+X_GeTe_2_ fashion.

## Teaser

We report the tunable 2D ferromagnetism above-room-temperature in wafer-scale Fe_3+X_GeTe_2_ films and demonstrate magnetic tunneling junction arrays using Fe_3+X_GeTe_2_ films.

## INTRODUCTION

Since the discovery of van der Waals two-dimensional (2D) materials, especially graphene [1], such 2D crystals have been widely extended to transition metal dichalcogenides [2] and 2D superconductors [3]. More recently, 2D magnets have attracted enormous attention because of the emergence of ferromagnetism in the monolayer limit [4,5]. Novel theoretical proposals and experiments in magnetic tunability and spintronic devices have been reported. Theoretically, moiré skyrmions [6], the nodal-line property [7], the quantum anomalous Hall effect [8] and the ‘magic angle’ effect on magnetism [9,10] have been proposed in 2D magnets and their heterostructures. Magneto-band-structure effect [11], described as the electronic band structure modified by magnetization directions, has also been predicted in 2D van der Waals ferromagnetic materials for the realization of giant magnetoresistance. Experimentally, the rapid exploration of new 2D ferromagnets provides a fertile ground for exotic magnetic properties, for instance, Curie temperature (*T*_C_) and coercive field (*H*_C_) tunability via gate voltage [12,13], magnon-assisted tunneling [14] and giant magnetoresistance [15–17]. In spite of the tremendous progress made in the CrX_3_ system, its *T*_C_ remains below 60 K and the exploration of high *T*_C_ materials becomes particularly appealing. Fe_3_GeTe_2_ exhibits a relatively high *T*_C_ of ∼220 K in the bulk state with a strong perpendicular magnetic anisotropy [18]. In exfoliated Fe_3_GeTe_2_ nanoflakes with a sample size in the order of micrometers, *T*_C_ achieves a high modulation even up to room temperature via ionic liquid gating [19]. Characterized by magnetotransport and angle-resolved-photoemission spectroscopy, the bulk Fe_3_GeTe_2_ is proposed to be a ferromagnetic nodal-line semimetal [7] that promises more exotic properties like magnetically tunable nodes [20,21]. An intriguing proposal, with regard to such materials, is to realize the quantized anomalous Hall effect at significantly higher temperatures in the monolayer limit [22,23]. However, the approach to achieving controllable growth with large-scale functioning devices and high-*T*_C_ ferromagnetic order remains elusive to date.

Chemical doping, via intentionally introducing impurities into parent materials, has been established as a direct yet effective approach to modulating and functionalizing the intrinsic electronic properties of 2D materials [24,25]. Doped transition metal dichalcogenides exhibit tunable electronic and optoelectronic properties [26–29]. Through Cr doping, the quantized anomalous Hall effect at millikelvin temperatures was discovered in Cr-doped (Bi, Sb)_2_Te_3_ films [30]. Dilute magnetic semiconductors, such as (Ga, Mn)As, yield a large modulation of *T*_C_ with different Mn compositions [31,32]. Scenarios of nitrogen-decorated NbSe_2_ nanosheets show the coexistence of ferromagnetism and superconductivity [33]. In Fe_3−X_GeTe_2_ bulk crystals [34] and films made by molecular-beam epitaxy (MBE) [35], the ferromagnetic behavior of *T*_C_ undergoes a monotonically decreasing trend with the reduction of the Fe composition. Nevertheless, the atom-doping-engineered *T*_C_ in 2D materials remains lower than 250 K, and further effective methods for magnetism modulation and the investigation into the underlying mechanism are indispensable.

Here, we employ a precise control of element flux in MBE to directly accomplish a *T*_C_ of 320 K in wafer-scale Fe_3+1.80_GeTe_2_ films. Aberration-corrected scanning transmission electron microscopy (STEM) investigations confirm the well-preserved layered structure in Fe-rich films. The angle-dependent anomalous Hall effect (AHE) evidences the persistent perpendicular magnetic anisotropy up to its *T*_C_ of 320 K, which is consistent with that deduced from zero-field-cooled (ZFC) and field-cooled (FC) susceptibility results (*T*_C_ ∼ 316.1 K) and X-ray magnetic circular dichroism results (XMCD, *T*_C_ ∼ 313.3 K). The *T*_C_ of the Fe_3+X_GeTe_2_ films is found to be strongly dependent on the X value, which continuously increases from ∼185 K (X = −0.25) to 320 K (X = 1.80) followed by the decreasing behavior to 290 K at X = 2.80. Density functional theory (DFT) calculations confirm the ferromagnetic ground state of the bulk Fe_3_GeTe_2_ via a comparison with different antiferromagnetic states. Moreover, the calculations find that the doped interlayer Fe atoms contribute significantly to the *T*_C_ enhancement. Based on these high-quality thin films, Fe_3+0.76_GeTe_2_/MgO/Fe_3_GeTe_2_ magnetic tunneling junction (MTJ) arrays are fabricated and clear tunneling signals are distinguished with a low-temperature tunneling magnetoresistance (TMR) ratio of ∼0.25%.

## F${\bf e} $_3+X_G${\bf e} $T${\bf e} $_2_ FILM SYNTHESIS

**Figure 1.**
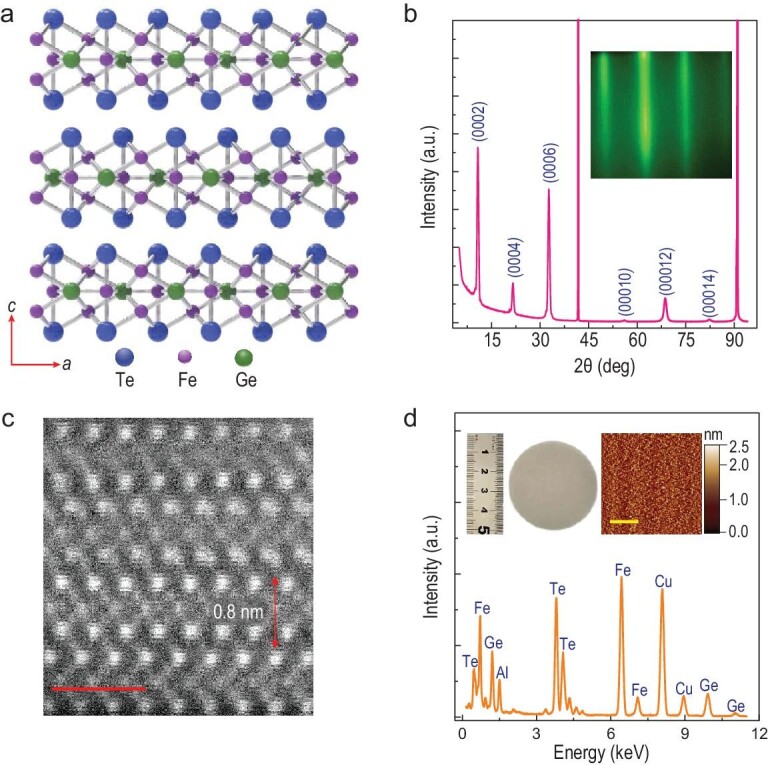
2D layered structure in Fe_3+X_GeTe_2_ thin films. (a) Fe_3_GeTe_2_ structure geometry. (b) XRD spectrum from Fe_3+0.18_GeTe_2_, with the peaks ascribed to (0002), (0004), (0006), (00010), (00012) and (00014) according to PDF# 75-5620. Inset, an RHEED pattern. (c) A cross section HAADF image of Fe_3+1.06_GeTe_2_. Layered structure with an interlayer distance of 0.8 nm is well-preserved in such Fe-rich films. The scale bar is 1 nm. (d) EDS for Fe_3+1.06_GeTe_2_. Left inset, a photograph of a 2-inch Fe_3+1.06_GeTe_2_ film. Right inset, an atomic force microscopy image taken from a 10 μm × 10 μm surface, showing the average surface roughness of 0.32 nm. The scale bar is 3 μm.

The layered Fe_3_GeTe_2_ compound has a hexagonal structure with the lattice parameters of *a *= 3.991(1) Å, *c *= 16.33(3) Å and a space group of P6_3_/mmc [36]. Figure 1a shows the projection view of the Fe_3_GeTe_2_ atomic structure along the [01–10] zone-axis, in which each layer consists of five sub-layers [36] with a Fe_3_Ge slab sandwiched between two neighboring Te layers with the corresponding nominal valence state of (Te^2–^)(Fe^3+^)[(Fe^2+^)(Ge^4–^)](Fe^3+^)(Te^2–^). By controlling the growth temperature and the flux of each element, high-crystalline Fe_3+X_GeTe_2_ films can be successfully grown by MBE. Figure 1b is an X-ray diffraction (XRD) pattern taken from a representative film, from which diffraction peaks can be ascribed to a series of {0002} planes (PDF# 75–5620). Its inset displays a streaky *in-situ* reflection high-energy electron diffraction (RHEED) pattern, indicative of a layer-by-layer growth mode for Fe-doped Fe_3+X_GeTe_2_ films (also displayed in Fig. S1). Figure 1c is a STEM-high angle annular dark-field (HAADF) image taken from a typical cross section of the film and shows the layered structure with an interlayer distance of ∼0.8 nm (close to the determined value for the stoichiometric Fe_3_GeTe_2_ films [35,37]). Therefore, the layered structure and high crystalline quality in Fe-rich Fe_3+X_GeTe_2_ thin films are well preserved. Figure 1d shows the corresponding X-ray energy dispersive spectrometry (EDS) profile of the film, and the quantitative analysis suggests the composition of the epitaxial Fe_3+X_GeTe_2_ is Fe_3+1.06_GeTe_2_. The left inset is a photograph of a 2-inch Fe_3+1.06_GeTe_2_ film, and the right inset shows an average surface roughness of 0.32 nm in the area of 10 μm × 10 μm detected by atomic force microscopy.

## ROOM-TEMPERATURE FERROMAGNETISM IN F${\bf e} $_3+1.80_G${\bf e} $T${\bf e} $_2_ FILM

**Figure 2.**
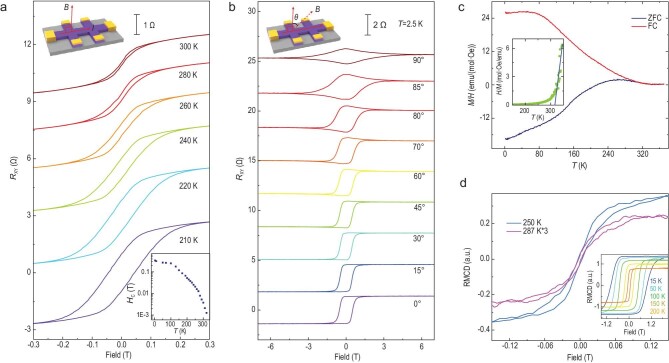
Out-of-plane ferromagnetic anisotropy of Fe_3+1.80_GeTe_2_ film with *T*_C_ of ∼320 K. (a) Temperature-dependent AHE under the perpendicular measurement geometry. Top inset, a schematic configuration of the perpendicular geometry between the sample surface and the magnetic field. Bottom inset, coercive field tracked from AHE. Up to 320 K, visible hysteresis can be distinguished, and vanishes at 330 K. *T*_C_ can be determined to be ∼320 K. (b) Angle-dependent AHE at 2.5 K. Because *H*_C_ increases with *θ* tilting from 0° to 90°, the easy axis is determined to be out-of-plane. Inset, the schematic geometry that defines the angle *θ*. (c) Zero-field-cooled (ZFC) and field-cooled (FC) susceptibility curves under a magnetic field of 200 Oe. *T*_C_ is determined to be 316.1 ± 2.6 K by the Curie-Weiss law as shown in the inset. The detailed estimation process is described in Supplementary Note S3. (d) Temperature-dependent polar RMCD curves. *H*_C_ and remanent magnetization decrease as the temperature increases, while ferromagnetic order still exists at 287 K.

To experimentally probe the high-*T*_C_ ferromagnetism in Fe_3+1.80_GeTe_2_ films, we carried out magnetotransport and *M-H* measurements. Unless specifically mentioned, hereafter, the thickness of Fe_3_GeTe_2_ films is ∼10 nm. The Hall effect for general ferromagnetic materials can be described as

$$\begin{eqnarray*}
{R_{xy}} = {R_H}B + {R_{AH}}M,
\end{eqnarray*}$$

where the Hall coefficient *R*_H_ stands for the ordinary Hall effect that is linearly dependent on the magnetic field (*B*), and the anomalous Hall effect *R*_AH_*M* comes from the magnetization (*M*) contribution. The AHE component can be obtained by subtracting the linear Hall resistance from the total Hall effect data, as illustrated in Fig. 2a. By increasing the temperature, the coercive field (*H*_C_) decreases correspondingly. Up to 300 K, the anomalous Hall resistance (*R*_XY_) still shows a hysteresis as the magnetic field scans back and forth; and eventually *H*_C_ vanishes at 330 K (Fig. 2a inset), based on which *T*_C_ is estimated to be ∼320 K. It should be noted that in exfoliated Fe_3_GeTe_2_, perpendicular magneto-crystalline anisotropy persists to monolayer even though *T*_C_ has been largely suppressed [19].

To characterize the Fe-doping effect on its magnetic anisotropy, the angle-dependent AHE at different temperatures is investigated. Here, the angle *θ* is defined as the angle between the magnetic field and the normal vector of the sample surface, as illustrated in the inset of Fig. 2b. At 2.5 K, the easy axis is confirmed to be along the out-of-plane direction with a perpendicular magnetic anisotropy due to the fact that the *H*_C_ increases simultaneously with the angle rotating from 0° to 90°, thus sharing the same anisotropy property as the stoichiometric Fe_3_GeTe_2_ [35]. This perpendicular anisotropy persists up to 320 K, as verified by the angle-dependent AHE at 270 K, 300 K and 320 K, shown in Fig. S7. Analyzed with the Stoner-Wohlfarth model [19,38], the perpendicular magneto-crystalline anisotropy energy density (*K*_u_) is estimated to be ∼1.08 × 10^7^ erg/cm^–3^ (Supplementary Note S2), which is comparable to that of the Fe_3_GeTe_2_ bulk crystals [38]. We have further explored the zero-field-cooled/field-cooled (ZFC-FC) magnetization curves for Fe_3+1.80_GeTe_2_ film (Fig. 2c, details in Supplementary Note S3), which exhibit different trends as the temperature decreases; they start to separate at ∼320 K. The variation of magnetization as a function of temperature is positively proportional to the magnetic susceptibility, which can be fitted by the Curie-Weiss law

$$
\begin{eqnarray*}
\chi = {\chi_0}{{+{\rm C}/({\rm T} -}}{{T}_C}),
\end{eqnarray*}$$

where ${\chi _0}\ $is a temperature-independent parameter resulting from the density of states at the Fermi energy level, and C is the Curie constant. The best fit to the experimental *FC* curve yields a *T*_C_ of 316.1 ± 2.6 K (Fig. 2c inset), consistent with the value tracked from the temperature-dependent AHE (Fig. 2a). The *M-H* curves at different temperatures are illustrated in Fig. S12a, where the coercive field of 40 Oe can be distinguished at 300 K.

Now the global room-temperature ferromagnetism in the millimeter-level flakes has been verified both by AHE and magnetization measurement. We further carried out the surface-sensitive polar reflective magnetic circular dichroism (RMCD) measurement where the focused laser spot was ∼3 μm to investigate its local magnetism. Figure 2d displays temperature-dependent RMCD measurement as a function of *B*. Consistent with the decreasing *H*_C_ and *R*_XY_ in the AHE measurements, the *H*_C_ and remanent magnetization decrease with the increasing temperature. It remains visible at 287 K and therefore confirms the enhanced ferromagnetism and the film uniformity. Combined with the persistent perpendicular magneto-crystalline anisotropy at various temperatures (Figs 2b and S7), this high *T*_C_ behavior in Fe_3+1.80_GeTe_2_ films can be confirmed and the presence of either Fe films or magnetic clusters can be unambiguously excluded [39–41] (Supplementary Note S2). In addition, XMCD results are also presented next to safely exclude these extrinsic effects.

**Figure 3.**
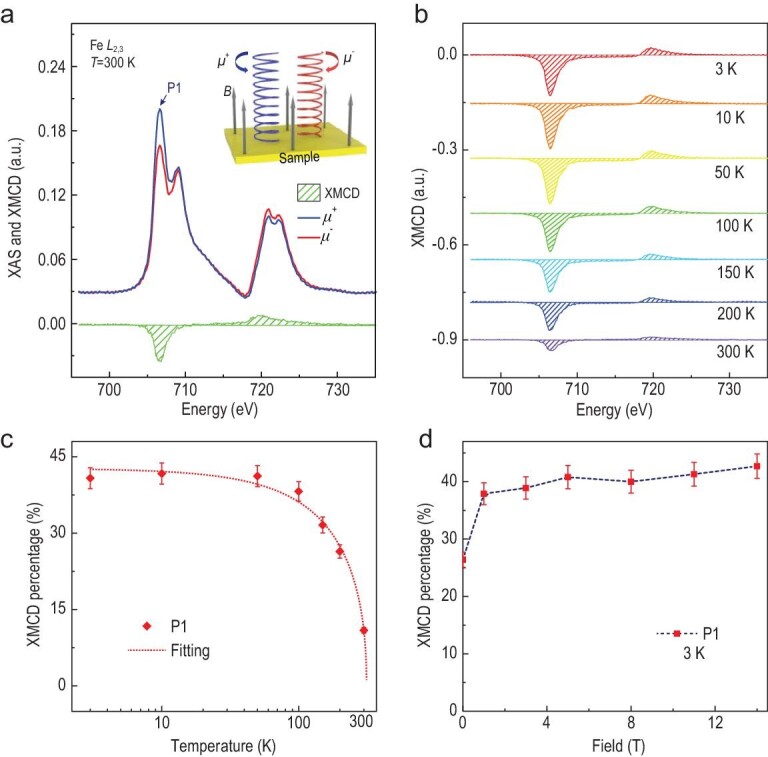
XAS spectra and XMCD signals of an Fe_3+1.80_GeTe_2_ sample at Fe *L*_2,3_ edges. (a) Room-temperature XAS and XMCD spectra of Fe *L*_2,3_ edges at the field of 5T. The agreement with the XAS of Fe_3_GeTe_2_ bulks [43] in the spectra shape further confirms the intrinsic room-temperature ferromagnetism. The two peaks at the Fe *L*_3_ edge suggest two sites of Fe, with the XMCD percentages calculated to be (10.90 ± 1.0)% and (1.47 ± 0.1)%, respectively. Inset, schematic of the XMCD experiments. (b) Temperature-dependent XMCD of Fe *L*_2,3_ edges where the spectra at different temperatures have vertical offsets for clarity. The magnetic field is fixed at 5T. (c) XMCD percentage versus temperature. As the temperature rises, the XMCD percentage decreases continuously. The dashed lines represent the XMCD percentages fitting to the empirical equation ${(1 - T/{T_C})^\gamma }$. *T*_C_ values are determined to be 313.3 ± 9.5 K, which further confirms the above-room-temperature ferromagnetism in Fe_3+1.80_GeTe_2_. (d) Field-dependent XMCD percentage, showing a large remanent XMCD percentage of 26.7% at zero-field.

The element-specific XMCD was further performed to probe the localized magnetism. Left (blue) and right (red) circularly polarized X-rays, denoted as *μ*^+^ and *μ*^–^, were used to resolve the XMCD signals, which was in parallel to the external magnetic field and in the normal incidence with respect to the sample surface (Fig. 3a inset). The XMCD signals were obtained by taking the difference of the X-ray absorption spectroscopy (XAS) spectra, i.e. ${\textit{XMCD}} = {\mu ^ - } - {\mu ^ + }$. The XAS spectra obtained in total-fluorescence yield mode were subtracted by a two-step function [42] and a strong XMCD signal was acquired at 300 K, as shown in Fig. 3a. The agreement with the XAS of Fe_3_GeTe_2_ bulk crystals [43] in the spectra shape further confirms its intrinsic high *T*_C_ ferromagnetism in the doped films, possessing two similar sites of Fe with such crystals [44–47]. The lower the temperature, the stronger the observed XMCD intensity (Fig. 3b). Here, to estimate the magnetic order, the XMCD percentage *β*, defined as the intensity ratio of XMCD to XAS in the equation $\beta = \frac{{({\mu ^ - } \ -\ {\mu ^ + })}}{{({\mu ^ - } \ +\ {\mu ^ + })}}$, is utilized as a parameter, which is calculated to be (10.9 ± 1.0)% and (1.5 ± 0.1)% for the two peaks at *L_3_* edge. As the critical peak on the left side of Fe *L*_3_ edge (marked as P1) gives the strongest dichroism, which suggests a larger magnetic contribution, we focus on P1 during the XMCD analyses. As shown in Fig. 3c, the temperature-dependent XMCD percentages can be fitted with an empirical function ${(1 - T/{T_C})^\gamma }$to extract the Curie temperature [48,49], based on which *T*_C_ is determined to be 313.3 ± 9.5 K. These results confirm our findings regarding the above-room-temperature ferromagnetism in Fe_3+1.80_GeTe_2_. In addition, solid ferromagnetism can be identified with a strong remanent XMCD percentage of 26.4% under zero magnetic field at 3 K (Fig. 3d).

## TUNABLE MAGNETISM AND THEORETICAL CALCULATION

**Figure 4.**
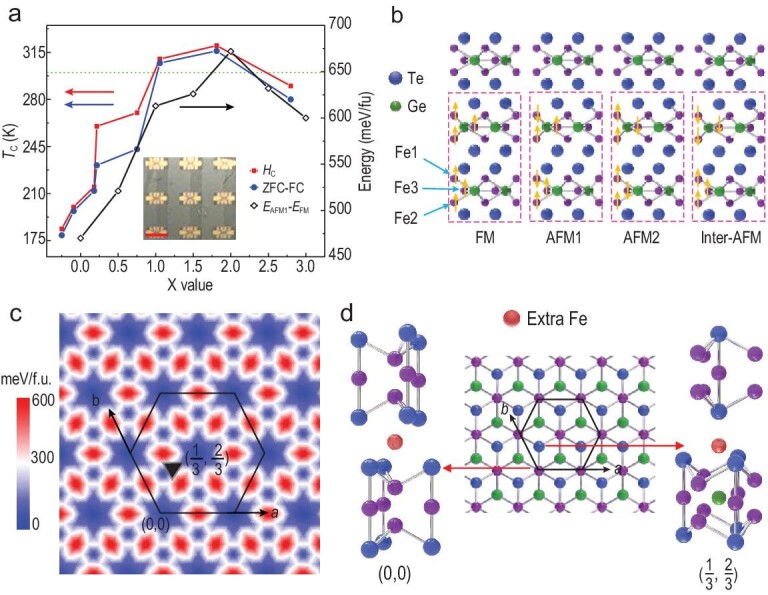
*T*_C_ modulation in Fe_3+X_GeTe_2_ film via Fe composition and DFT calculations. (a) *T*_C_ versus X ratio, reaching a peak value of 320 K at X = 1.80. Inset: an optical image of MTJ device arrays. The scale bar is 2 μm. (b) Schematic diagrams for the four defined magnetic states, the orange arrows illustrating the spin direction of each Fe1, Fe2 and Fe3 atom. (c) Relative total energies map of an extra Fe atom in the different interlayer positions of Fe_3_GeTe_2_ calculated by LSDA + U. There are three most stable sites at (0,0), (1/3,2/3) and (2/3,1/3). (d) Local structure of an extra Fe at (0,0) or (1/3,2/3) in bulk Fe_3_GeTe_2_.

In stark contrast to the continuously-decreased *T*_C_ in Fe-deficient Fe_3−δ_GeTe_2_ samples [34,35] where the Fe composition deviates negatively (δ < 0.3) from Fe_3_GeTe_2_, here we present a large enhancement of the ferromagnetic order in Fe_3+X_GeTe_2_ films by systematically tuning the X value from −0.25 (Fe-deficient) to 2.80 (Fe-rich). As illustrated in Fig. 4a, *T*_C_ initially increases with the increasing Fe doping, reaches a maximum value of 320 K at X = 1.80 and finally drops to 290 K in Fe_3+2.80_GeTe_2_. This *T*_C_ behavior is a prominent extension to that of the Fe-deficient Fe_3−δ_GeTe_2_ samples. Utilizing the high-*T*_C_ and large-scale thin films, we have built MTJ device arrays (Fig. 4a inset) with an Fe_3+0.76_GeTe_2_/MgO/Fe_3_GeTe_2_ device structure (Supplementary Note S4). Clear tunneling magnetoresistance signals can be detected as the magnetic field scans back and forth. However, the tunneling magnetoresistance ratio is still low (∼0.25%), which calls for further improvements on the crystalline quality of MgO.

In order to provide insight into the observed room-temperature ferromagnetic behavior in Fe_3+X_GeTe_2_ films, we performed DFT calculations within the LSDA + U framework to understand the bulk Fe_3_GeTe_2_ and its doping effect (Supplementary Note S5 and Fig. S17). We chose four different magnetic states, namely, the FM, AFM1, AFM2 and inter-AFM states, as illustrated in Fig. 4b. For the bulk, the LSDA + U calculations using the experimental lattice parameters confirm the FM ground state as summarized in Table 1. It is more stable than the inter-AFM state by 18 meV per formula unit (f.u.), indicating a relatively weak ferromagnetic interlayer coupling associated with the van der Waals bonding of the 2D material. However, due to the metallic behavior of Fe_3_GeTe_2_, the intralayer itinerant FM is quite strong. Compared with the FM ground state, the AFM1 state lies much higher in energy (by 300 meV/f.u.). This energy cost is due to the suppressed electron itinerancy in the AFM1 state (with one AFM Fe1-Fe3-Fe1 zigzag channel, see Fig. 4b) and the corresponding reduced kinetic energy gain. If two AFM zigzag channels (Fe1-Fe3-Fe1 and Fe2-Fe3-Fe2, see Fig. 4b) appear as in the AFM2 state, the energy cost is calculated to be 624 meV/f.u., being nearly doubled compared with the AFM1-FM energy difference with the change of one magnetic channel. Therefore, in our calculations, we employed the AFM1-FM energy difference to characterize the stability of the FM ground state and to trace the varying FM stability with the changing Fe concentrations.

**Table 1.** Relative total energy (meV/f.u.) and local spin moments (μ_B_) of different magnetic states calculated by LSDA + U for bulk Fe_3_GeTe_2_.

Magnetic state	Δ*E* (meV/f.u.)	Fe1 (μ_B_)	Fe2 (μ_B_)	Fe3 (μ_B_)
FM	0	2.74	2.74	1.96
AFM1	300	−2.74	2.62	1.77
AFM2	624	2.88	2.88	−1.64
Inter-AFM	18	2.72	2.72	1.95

Owing to the van der Waals layered structure of Fe_3_GeTe_2_, the additional Fe atoms most probably lie in the interlayer interstitial region. We use LSDA + U calculations to search the stable interlayer interstitial positions by optimizing the *c*-axis lattice parameter and atomic z coordinates. Our calculations find that, for a doped Fe atom, there are three most stable interlayer occupation positions on the 1 × 1 plane, (0,0), (1/3,2/3) and (2/3,1/3), which have almost the same potential well depth, as seen in Fig. 4c. This finding explains why the Fe concentration in Fe_3+X_GeTe_2_ can experimentally be largely enhanced.

To study the impact of the doped interlayer Fe atoms on the magnetism of Fe_3+X_GeTe_2_, we first compare the two cases of Fe_3+0.5_GeTe_2_ with one doped Fe atom on either the (0,0) or (1/3,2/3) position (Fig. 4d), using the LSDA + U calculations including a full atomic relaxation. The AFM1-FM energy difference is calculated to be 530 and 521 meV/f.u., respectively, showing insignificant site dependence of the FM strength in Fe_3+0.5_GeTe_2_ on the interlayer Fe positions. We then simulate Fe_3+X_GeTe_2_ (X = 0.5–3) by adding the interlayer Fe atoms in the AB stacking Fe_3_GeTe_2_ unit cell one by one, at A(1/3,2/3), B(2/3,1/3), A(2/3,1/3), B(1/3,2/3), A(0,0) and B(0,0), to minimize the interlayer Fe–Fe coordinations in each case. As seen in Fig. 4a, upon increasing the interlayer Fe concentrations, the calculated AFM1-FM energy difference increases from 470 meV/f.u. (after atomic relaxation) for the stoichiometric Fe_3_GeTe_2_ to the maximal 670 meV/f.u. for X = 2 and then drops to 600 meV/f.u. for X = 3. The maximal enhancement of the FM strength by ∼40% at the optimal concentration X = 2 agrees well with our experimental findings. This composition-dependent *T*_C_ in Fe_3+X_GeTe_2_ films correlates with the electron doping effect which enhances the itinerant FM up to an optimal doping level (Supplementary Note S6).

## CONCLUSION

In summary, we have demonstrated a direct doping approach in MBE growth to achieve high-*T*_C_ 2D ferromagnetic Fe_3+X_GeTe_2_ films beyond room temperature. Through systematically tuning the Fe composition, *T*_C_ experiences an efficient modulation from 185 K to 320 K, which arrives at the peak value of 320 K at Fe_3+1.80_GeTe_2_, validated by the temperature-dependent XMCD measurements. We further demonstrated large-scale MTJ device arrays based on Fe_3+X_GeTe_2_ films. Moreover, our DFT study suggests that the doped interlayer Fe atoms provide a strong tunability to the magnetic order, achieving the optimal enhancement of FM strength by 40% at X = 2. Therefore, this study opens an avenue to a significant enhancement of the *T*_C_ in emerging 2D ferromagnetic Fe_3+X_GeTe_2_ films, which may facilitate their practical application in spintronic devices.

## METHODS

### Thin film synthesis and characterization

Fe_3+X_GeTe_2_ thin films were synthesized on (0001)-sapphire in a Perkin Elmer 430 MBE system (base vacuum: ∼2.5 × 10^−9^ Torr). The substrates were firstly cleaned using a standard process, and before the growth, substrates were annealed at 600°C for 30 minutes, which was then cooled to the target temperature of 340°C. The growth temperatures for Ge-cell and Te-cell were 1020°C and 285°C, and the Fe composition was tuned via varying the Fe-cell temperature. The crystal oscillator was used to measure each element's flux. XRD results were measured in a Bruker D8 Discover facility and transmission electron microscope measurements were performed using JEOL JEM-ARM 200F and FEI Titan G2 systems.

### Electrical and magnetization measurement

Magnetotransport results were collected by SR830 in the Physical Properties Measurement System (PPMS) and the devices were in the six-Hall-bar geometry. The magnetization measurements were accomplished by DC-Superconducting-Quantum-Interface-Devices (SQUID) by Quantum Design.

### RMCD and XMCD measurements

RMCD measurements were performed in a closed-cycle helium cryostat with measurable temperature ranges from 15 to 287 K. A 633 nm HeNe laser with the power of ∼0.3 μW and the focused beam spot of 3 μm was in the normal incidence onto the sample. A lock-in amplifier was utilized to acquire the RMCD signals. XMCD measurements at Fe *L*_2,3_ edge were performed on beamline I10 at the Diamond Light Source.

### DFT calculation

DFT calculations were processed using the Vienna *ab initio* Simulation Package (VASP) [50,51]. Local density approximation to the exchange-correlation function was used [52], which has previously been shown to describe the structural properties of Fe_3_GeTe_2_ well [53]. A plane wave cut-off of at least 400 eV was employed. The Brillouin zone was sampled using an 8 × 8 × 3 k-point mesh. The ionic potentials, including the effect of core electrons, were described by the projector augmented wave method [54]. The atomic relaxations were implemented until the Hellmann-Feynman force on each atom was smaller than 0.01 eV/Å. We used the experimental lattice constants with atomic relaxations to study the magnetism of Fe_3+X_GeTe_2_. In addition to LSDA, the LSDA plus Hubbard U (LSDA + U) method was employed [51], and we chose U = 3.5 eV (and Hund exchange J = 0.9 eV) for the Fe 3d electrons to calculate the magnetic properties. The calculation details are shown in Supplementary Note S6.

## Supplementary Material

nwab117_Supplemental_File
