# Supplementary material for: Tuning 2D magnetism in Fe3+XGeTe2 films by element doping
Source: Natl Sci Rev. 2021 Jul 2;9(6):nwab117. doi: 10.1093/nsr/nwab117 (PMC9270067; doi:10.1093/nsr/nwab117)
Supplement: nwab117_Supplemental_File [file nwab117_supplemental_file.pdf]

Supplementary Information for

Tuning 2D magnetism in $Fe_{3+X}GeTe_2$ films by element doping

Shanshan Liu[1,2#], Zihan Li[1,2#], Ke Yang[3,4#], Enze Zhang[1,2], Awadhesh Narayan[5], Xiaoqian Zhang[6], Jiayi Zhu[7], Wenqing Liu[8], Zhiming Liao[9,10], Masaki Kudo[11], Takaaki Toriyama[11], Yunkun Yang[1,2], Qiang Li[1,2], Linfeng Ai[1,2], Ce Huang[1,2], Jiabao Sun,[8] Xiaojiao Guo[12], Wenzhong Bao[12], Qingsong Deng[10], Yanhui Chen[10], Lifeng Yin[1,2,13], Jian Shen[1,2,13], Xiaodong Han[10], Syo Matsumura[11,14], Jin Zou[9,15], Yongbing Xu[6], Xiaodong Xu[7], Hua Wu[1,4,13*], Faxian Xiu[1,2,13,16*]

[1]State Key Laboratory of Surface Physics and Department of Physics, Fudan University, Shanghai 200433, China

[2]Institute for Nanoelectronic Devices and Quantum Computing, Fudan University, Shanghai 200433, China

[3]College of Science, University of Shanghai for Science and Technology, Shanghai, 200093, China

[4]Laboratory for Computational Physical Sciences (MOE), Fudan University, Shanghai 200433, China

[5]Solid State and Structural Chemistry Unit, Indian Institute of Science, Bangalore 560012, India

[6]School of Electronic Science and Engineering, Nanjing University, Nanjing 210093, China

[7]Department of Physics, University of Washington, Seattle, WA 98195-1560, USA.

[8]Department of Electronic Engineering, Royal Holloway University of London, Egham TW20 0EX, United Kingdom

[9]Materials Engineering, The University of Queensland, Brisbane QLD 4072, Australia

[10]Beijing Key Lab of Microstructure and Property of Advanced Materials, Institute of Microstructure and Properties of Advanced Materials, Beijing University of Technology, Beijing 100124, China

[11]The Ultramicroscopy Research Center, Kyushu University, Fukuoka 819-0395, Japan

[12]State Key Laboratory of ASIC and System, School of Microelectronics, Fudan University, Shanghai 200433, China

[13]Collaborative Innovation Center of Advanced Microstructures, Nanjing 210093, China

[14]Department of Applied Quantum Physics and Nuclear Engineering, Kyushu University, Fukuoka 819-0395, Japan

[15]Centre for Microscopy and Microanalysis, The University of Queensland, Brisbane QLD 4072, Australia

[16]Shanghai Research Center for Quantum Sciences, Shanghai 201315, China

[#] These authors contributed equally to this work

[*]Correspondence and requests for materials should be addressed to F.X. (E-mail: Faxian@fudan.edu.cn) and H.W. (wuh@fudan.edu.cn).

Note S1. Characterizations of $Fe_{3+X}GeTe_2$ films

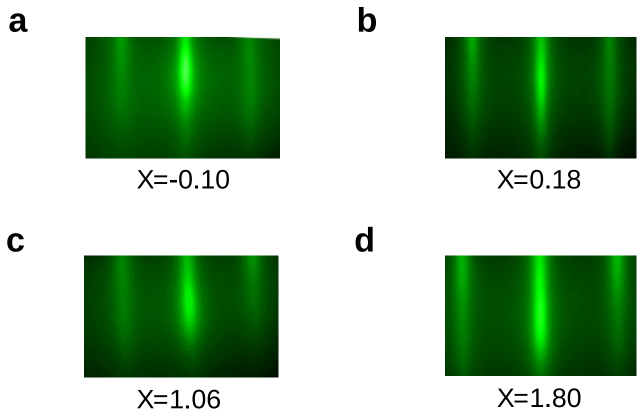

Figure S1. The evolution of the RHEED patterns with different Fe compositions in $Fe_{3+X}GeTe_2$ films. The streaky RHEED patterns suggest a smooth surface.

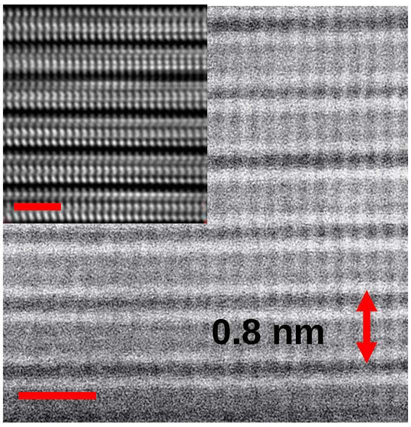

Figure S2. A cross-section HAADF image of $Fe_{3+1.06}GeTe_2$. Layered structure with the inter-layer distance of 0.8 nm is well-preserved. The scale bar is 1 nm.

Note S2. The AHE and magnetic anisotropy in $Fe_{3+X}GeTe_2$ films

The composition of the $Fe_{3+X}GeTe_2$ films is carefully adjusted to modulate the magnetic properties. To have a comprehensive study on our samples, we have carried out magnetotransport measurements on $Fe_{3+X}GeTe_2$ samples including angle-dependent and temperature-dependent AHE, SQUID, and XMCD experiments. Further, we built wafer-scale $Fe_{3+0.76}GeTe_2$/MgO/Fe_3GeTe_2 MTJ device arrays with clear tunneling signals.

The temperature-dependent AHE experiments were carried out to determine the Curie temperature (T_C), as shown in Figures S3-S5 in the sequence from the low Fe doping to the high one. With the increase of the temperature, the coercive field (H_C) becomes smaller and vanishes at T_C, based on which T_C is determined to be ~ 200 K (X= -0.10), and 215 K (X= 0.18), as shown in Figure S3. By increasing the Fe composition, T_C increases and reaches a peak of 320 K at X= 1.80, after which it follows a declining trend (Figure 4a).

Magnetic anisotropy of the $Fe_{3+X}GeTe_2$ films was investigated by angle-dependent AHE measurements. Here, we define three parameters, θ, θ_H, θ_M, and the schematic diagram is displayed in Figure S6. θ represents the angle between the external field and the normal vector of the Hall-bar device. θ_H is the angle between the external field and the Hall-bar device. θ_M denotes the angle between the sample magnetization direction and the device. Under a perpendicular magnetic field (θ=0°, θ_H=90°), the square-shape hysteresis loop suggests an out-of-plane magnetic anisotropy. With the magnetic field tilting slowly to the in-plane, the magnetization direction is gradually forced to be along the in-plane direction at θ_H=90°.

As shown in Figure 2b, for $Fe_{3+X}GeTe_2$ (X=1.80), at 2.5 K the easy axis is perpendicular to the sample surface as H_C becomes larger with the angle θ changing from 0° to 90°. This low-temperature perpendicular magnetic anisotropy is consistent with the stoichiometric Fe_3GeTe_2 bulk [1], exfoliated nanoflakes [2]. In order to exclude some possible origins, like Fe thin film, the angle-dependent AHE experiments up to its T_C of 320 K were conducted (Figure S7). H_C becomes larger at 90° when compared to that at 0°, confirming the similar perpendicular anisotropy to the one at 2.5 K. Through this, the perpendicular magnetic anisotropy is confirmed to persist to the Curie temperature.

Will this perpendicular magnetic crystalline anisotropy persist in heavily Fe-doped $Fe_{3+X}GeTe_2$ films? Here, as shown in Figure S9, for $Fe_{3+2.80}GeTe_2$ (the highest doping ratio) the easy-axis is all along the out-of-plane direction from 2.5 K to 285 K as H_C is the smallest for the out-of-plane geometry. In thin Fe films, however, with the thickness increasing, the magnetic anisotropy will change from out-of-plane to in-plane direction at the boundary of 6.5-monolayer [3,4]. For Fe clusters, the magnetic properties including ferromagnetic, antiferromagnetic and paramagnetic are dependent on the size and dwell time during growth [5]. And therefore, this out-of-plane easy-axis excludes possible origin from enriched Fe clusters or films, favoring its intrinsic ferromagnetic phenomenon.

We further explored the magnetic anisotropy energy evolution in the $Fe_{3+X}GeTe_2$ samples. To eliminate the domain effect, we analyzed the Hall data under a high magnetic field of 9 T at which all the anomalous Hall resistances are saturated. We extracted θ_M, as a function of θ_H, using the function of

$$\theta_M(\theta_H) = \sin^{-1}\left(\frac{R_H(\theta_H)}{R_H(\theta_H = 90°)}\right).$$

The magnetic anisotropy energy density is extracted by fitting θ_M, θ_H data with the Stoner-Wohlfarth model [1,6]. The total energy can be written as [6],

$$E = K_u \sin^2(\theta_M) - \mu_0 H M_s \cos(\theta_H - \theta_M),$$

where K_u is the magnetic anisotropy energy density, and M_S is the saturation magnetization per volume. In the equilibrium state:

$$\frac{\partial E}{\partial \theta_M} = 2K_u \sin(\theta_M)\cos(\theta_M) + \mu_0 H M_S \sin(\theta_H - \theta_M) = 0.$$

This equation describes the relationship between θ_M and θ_H, which can be used to fit the experimental data, as shown in Figure S10. Taking the M_S value of 1.8μ_B per Fe atom [8], we find that K_u exhibits a rising trend with the increase of the Fe composition which is deduced to be 4.65×10^6, 8.53×10^6, 1.08×10^7, and 1.90×10^7 erg cm^{-3} for $Fe_{3+X}GeTe_2$ films with X of -0.10, 1.06, 1.80, and 2.80, respectively, in a reasonable agreement with the value of bulk Fe_3GeTe_2 crystals [8].

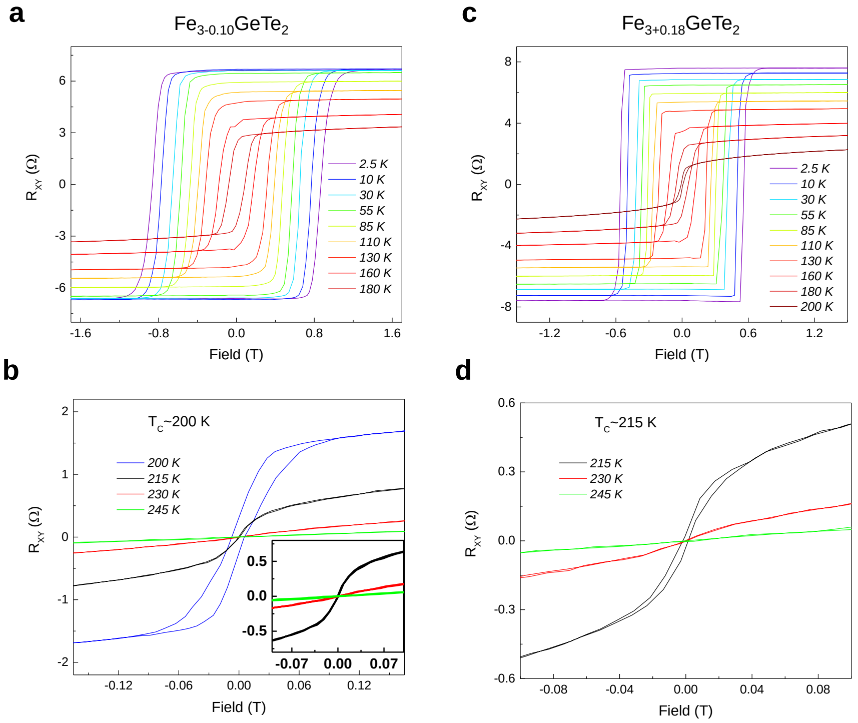

Figure S3. Temperature-dependent R_{XY} for $Fe_{3+X}GeTe_2$ films with X value of -0.10 (Figure S3a-b) and 0.18 (Figure S3c-d). T_C values are determined to be ~200 K and 215 K, respectively.

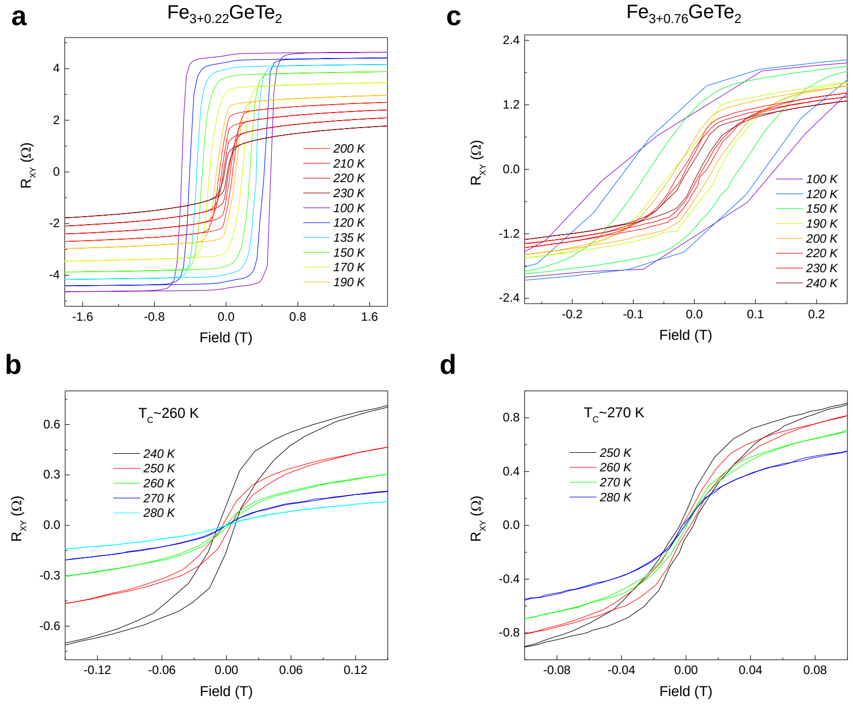

Figure S4. Temperature-dependent R_{XY} for $Fe_{3+X}GeTe_2$ films with the X value of 0.22 (Figure S4a-b) and 0.76 (Figure S4c-d). T_C values are determined to be ~260 K and 270 K, respectively.

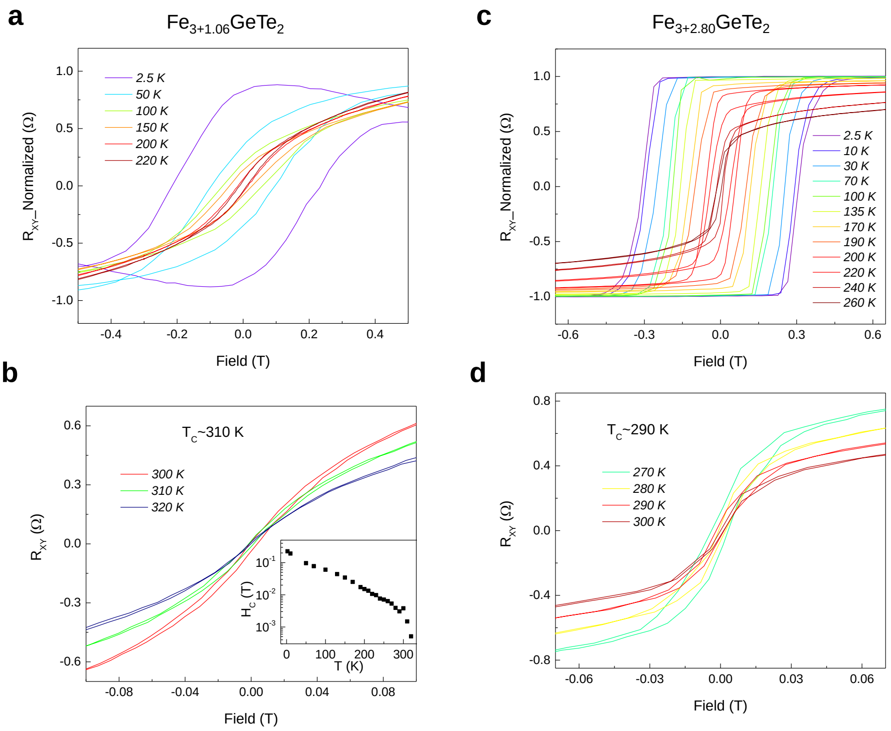

Figure S5. Temperature-dependent R_{XY} for $Fe_{3+X}GeTe_2$ films with the X= 1.06 (Figure S5a-b) and 2.80 (Figure S5c-d). T_C values are determined to be ~310 K and 290 K, respectively.

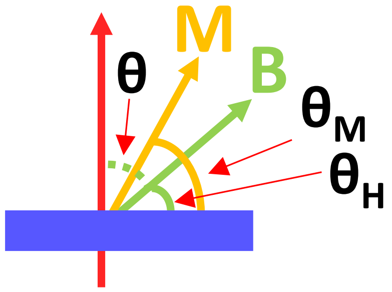

Figure S6. A schematic diagram for the angle definition of θ, θ_H, θ_M where $\theta_H = 90^{\circ}$-θ.

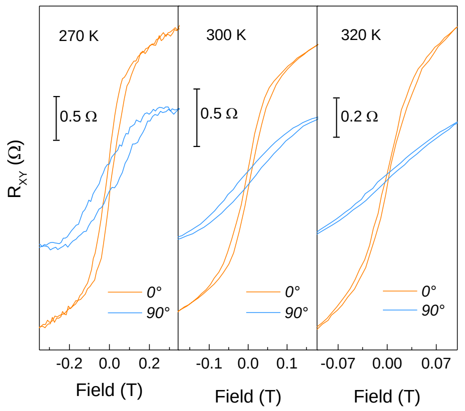

Figure S7. Angle-dependent AHE of $Fe_{3+1.80}GeTe_2$ film. Up to 320 K, the easy axis is

still along the out-of-plane direction.

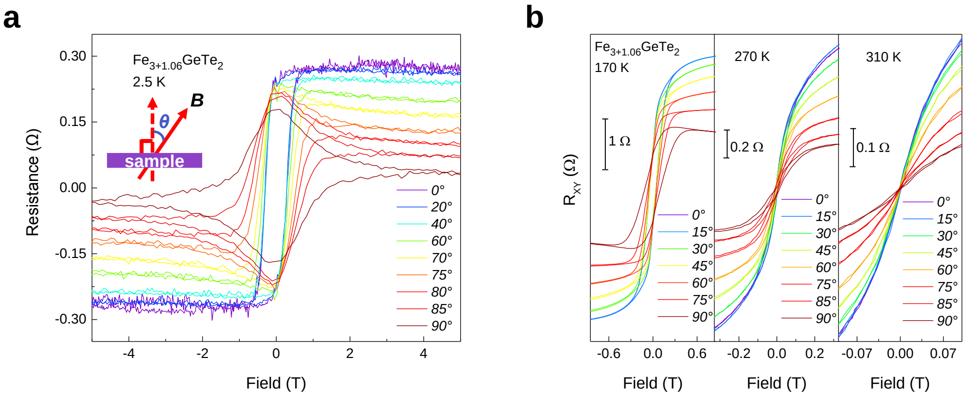

Figure S8. Angle-dependent AHE from $Fe_{3+1.06}GeTe_2$ film. The easy-axis is along out-of-plane direction from the low temperature of 2.5 K to its T_C of 310 K. T_C

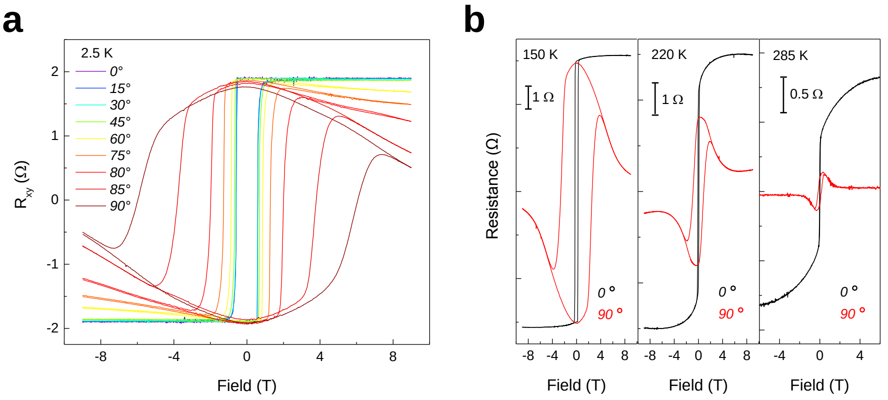

Figure S9. Angle-dependent AHE in $Fe_{3+2.80}GeTe_2$ film. The easy-axis is along out-of-plane from the low temperature of 2.5 K to 285 K.

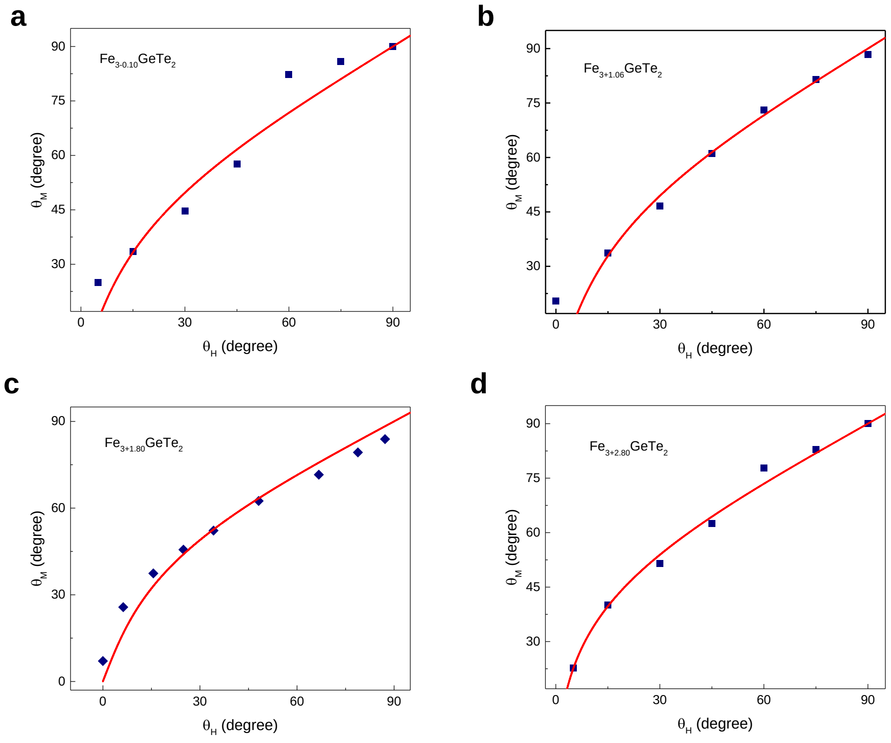

Figure S10. θ_M as a function of θ_H. Solid lines are fittings to the Stoner–Wohlfarth

model. Taking M_S of 1.8μ_B per Fe, the magnetic anisotropy energy density K_u is deduced to be 4.65×10^6, 8.53×10^6, 1.08×10^7, and 1.90×10^7 erg cm^{-3} erg cm^{-3} for the (a) $Fe_{3-0.10}GeTe_2$, (b) $Fe_{3+1.06}GeTe_2$, (c) $Fe_{3+1.80}GeTe_2$, and (d) $Fe_{3+2.80}GeTe_2$.

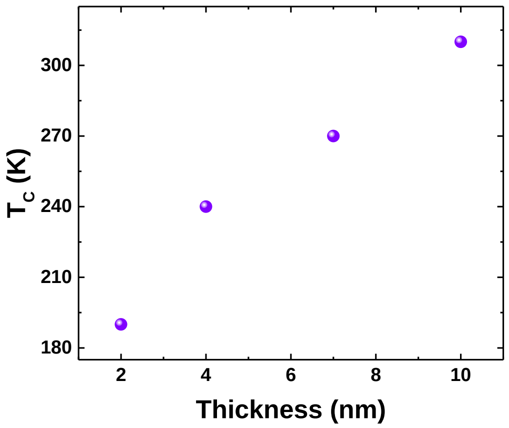

Figure S11. Thickness-dependent T_C for $Fe_{3+X}GeTe_2$ films with a fixed X value of 1.06. T_C exhibits a declined trend as the thickness reduces, which is similar to the dimensionality-effect on other 2D ferromagnetic materials.

Note S3. SQUID and XMCD results on $Fe_{3+X}GeTe_2$

Through magneto-transport measurements, the easy axis for the $Fe_{3+X}GeTe_2$ films is along the out-of-plane direction. The perpendicular geometry between the sample surface and the magnetic field is taken during the SQUID measurements.

The susceptibility and saturation magnetization for the $Fe_{3+X}GeTe_2$ films were estimated with the size of the experimentally-used sample of 2.5 mm×2 mm×10 nm. Based on the previously reported $Fe_{3-x}GeTe_2$ data [8] and our results, the volume of doped Fe_3GeTe_2 unit cell has a negligible change, so we use its standard value of 112.67 Å^3. The mole number and N_{Fe} can be estimated by

$$mole = \frac{V_{FGT}}{112.67\text{Å}^3}/N_A, \qquad \text{(I)}$$

$$N_{Fe} = \frac{V_{FGT}}{112.67\text{Å}^3} \times n_{Fe}, \qquad \text{(II)}$$

The susceptibility in the unit of (emu/(mol·Oe)) and magnetization in the unit of (μ_B/Fe) can be calculated as:

$$\chi = \frac{Moment\ (emu)}{mole}/\text{H (Oe)}, \qquad \text{(III)}$$

$$\text{M} = \frac{Moment\ (emu) * 10^{-3} (Am^2/emu)}{N_{Fe} * \mu_B}, \qquad \text{(IV)}$$

where N_A, n_{Fe}, H and μ_B are Avogadro constant, the Fe number in one unit-cell, the filed, and Bohr magneton, and 10^{-3} is the transformation coefficient between CGI and SI.

The magnetization of $Fe_{3+1.80}GeTe_2$ (temperature of 2.5 K) is calculated to be ~1.32 μ_B per Fe-atom (Figure S12b). In the various Fe-deficient polycrystalline and crystalline Fe_xGeTe_2 (x: 2.75~3.10) bulks, the magnetization is in the range of 1.0-1.4 μ_B (2 K) [8], and the reported magnetization of Fe_3GeTe_2 single crystals are 1.2 μ_B (5

K)[9], 1.4 μ_B (1.8 K) [10], 1.58 μ_B (5 K) [11], and 1.625 μ_B (zero temperature) [12]. Therefore, the saturation magnetization in our films agrees with this experimental value of bulk crystals.

In our static LSDA+U calculations, the magnetic moment is 2.48 μ_B per Fe in Fe_3GeTe_2 bulk (Table I), being well comparable with other calculated values[1,13] of 2-2.7 μ_B. Note that including our results, all the above experimental values[8,10,11] are smaller than the calculated static magnetic moment due to a strong spin fluctuation in this itinerant ferromagnet.

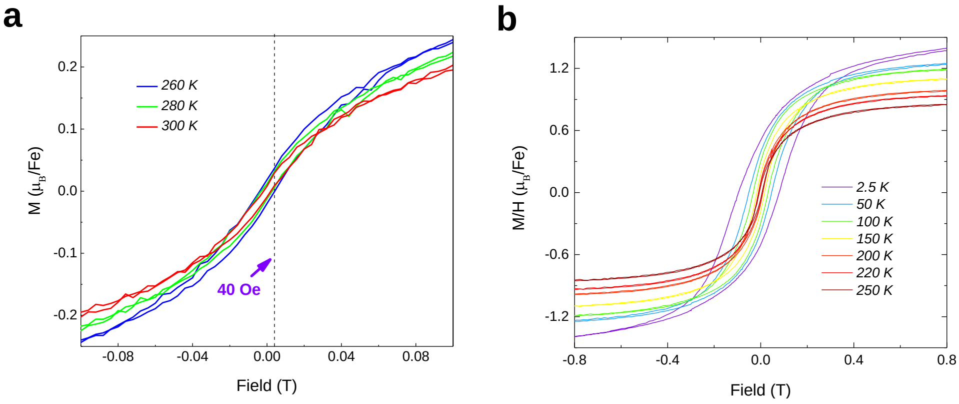

Figure S12. Field-dependent magnetization loops for $Fe_{3+1.80}GeTe_2$ at different temperatures. Hysteresis can still be observed at 300 K (room temperature).

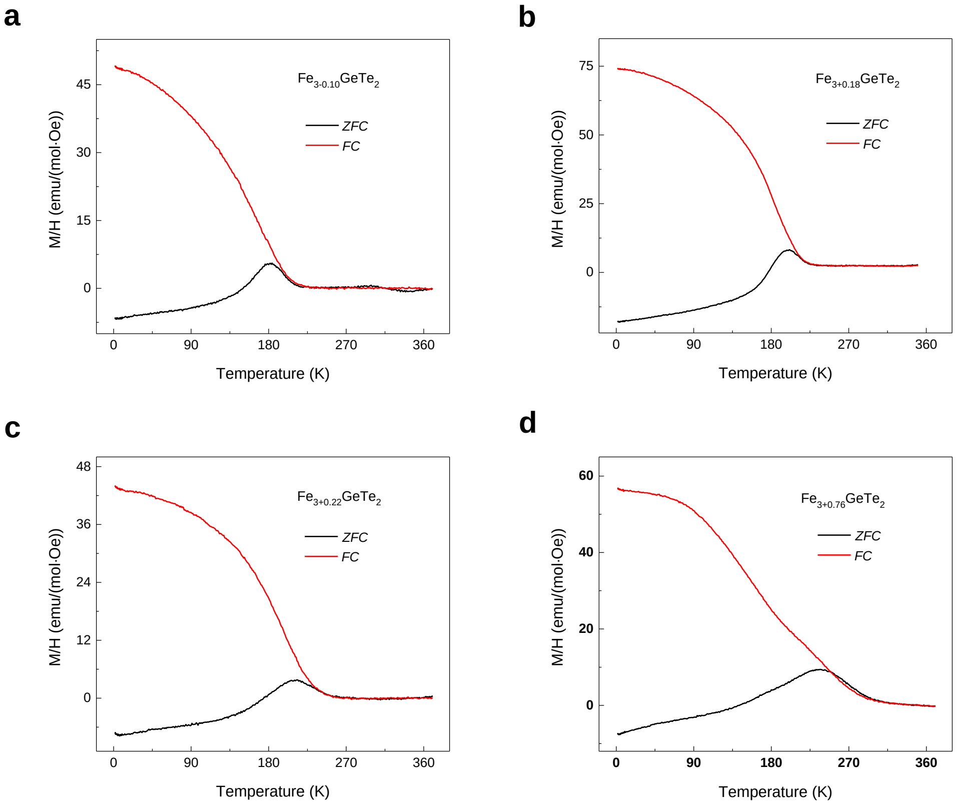

Figure S13. Zero-field-cooled and field-cooled (ZFC-FC) magnetic suscepbility results for $Fe_{3+X}GeTe_2$ with the X value ratio of (a) -0.10, (b) 0.18, (c) 0.22, and (d) 0.76. T_C is determined to be 197 K, 212 K, 231 K, and 243 K, respectively.

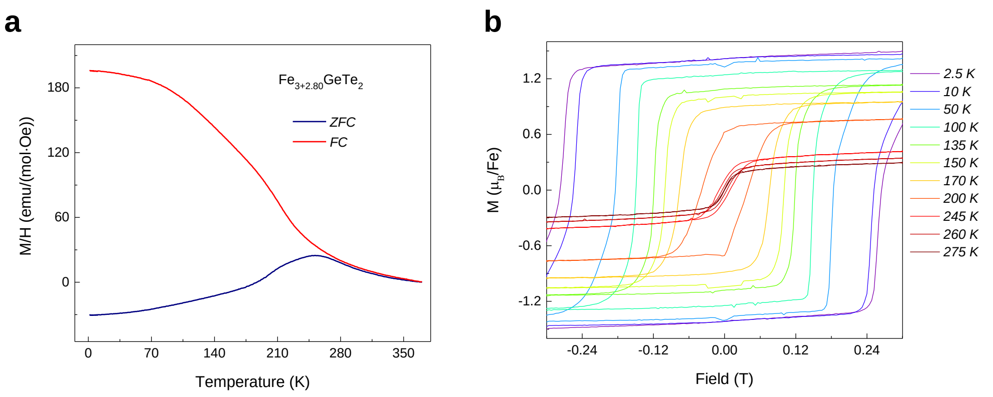

Figure S14. SQUID measurements for $Fe_{3+2.80}GeTe_2$. (a) ZFC-FC susceptibility curves, where T_C is determined to be ~280 K. (b) Magnetization hysteresis at different temperatures.

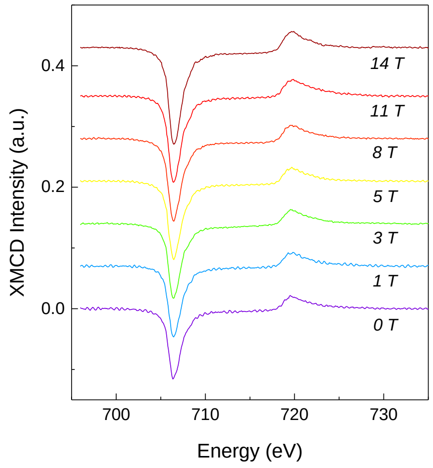

Figure S15. XMCD evolution for the $Fe_{3+1.80}GeTe_2$ sample versus the magnetic field, obtained at 3 K.

Note S4. MTJ device arrays based on $Fe_{3+0.76}GeTe_2$/MgO/ Fe_3GeTe_2

To fabricate magnetic tunneling devices, we use $Fe_{3+0.76}GeTe_2$ and Fe_3GeTe_2 as the top and bottom layers, respectively, which are separated by a 2-nm-thick MgO layer. For $Fe_{3+0.76}GeTe_2$, H_C is slightly smaller than that of stoichiometric Fe_3GeTe_2. Firstly, we grew Fe_3GeTe_2, MgO, and $Fe_{3+0.76}GeTe_2$ layers successively without taking the sample out of the chamber in order to get the furthest clean interface. We then performed the etching process twice to get the bottom Fe_3GeTe_2 exposed at one side (contact 2&5&6 area, top panel in Figure S16a) while another side keeps the same structure of $Fe_{3+0.76}GeTe_2$/MgO/ Fe_3GeTe_2 (contact 1&3&4 area, top panel in Figure S16a) and one contact deposition (bottom panel in Figure S16a). Finally, we have constructed wafer-scale MTJ arrays (Figure S16a). Displayed in Figure S16b, we can observe two-switching behavior in R_{xy} as the magnetic field scans back and forth (measured between contacts 3&4), accompanied by R_{xx} tunneling jumps successively (measured between contacts 1&2). The small H_C (~0.3 Tesla) belongs to the top $Fe_{3+0.76}GeTe_2$ layer while the large one at ~0.9 Tesla corresponds to the bottom Fe_3GeTe_2. However, at the narrow region of the magnetic field, like 0.3~1 Tesla

where the switching feature appears, we did not observe highly insulating R_{xx} (Figure S16c), which is possibly due to the non-uniform interface or the thick FGT layer. Here, ΔR is defined to characterize the R_{xx} switching ratio. As illustrated in Figure S16d, at 10 K, the tunneling ratio is estimated to be ~0.25% and with the temperature increasing the ratio decreases monotonously. This low efficiency possibly comes from the non-uniform MgO layer, which calls for further improvements in the MgO growth and the fabrication process.

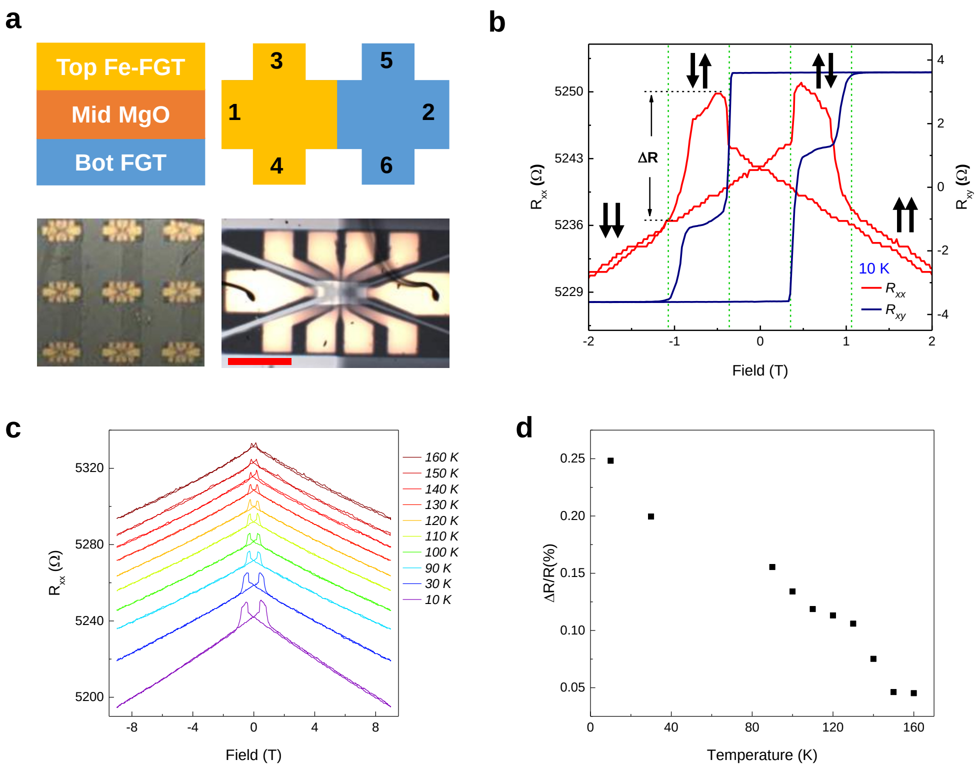

Figure S16. MTJ device arrays based on $Fe_{3+0.76}GeTe_2$/MgO/ Fe_3GeTe_2. (a) Schematic MTJ structures from side view and top view (top panel). The bottom panels are the large-scale MTJ arrays and a representative device that is achieved after etching and gold deposition processes. The thickness of MgO layer is ~2 nm and the scale bar is 0.5 μm. (b) R_{xx} and R_{xy} detected from 1&2 and 3&4 at 10 K (Figure S16a). Two-switching behavior in the R_{xy} curve suggests the spin of top and bottom FGT layers switches successively versus the magnetic field, which is consistent with the jumps in R_{xx}. The black arrows stand for the spin direction of the top and bottom magnetic layers, respectively. (c) Field-dependent R_{xx} at different temperatures. (d) Tunneling magnetoresistance ratio versus temperature.

Note S5. LSDA and LSDA+U calculations for Fe_3GeTe_2

Our LSDA calculations find that the optimized lattice constants of Fe_3GeTe_2 bulk, a=b=3.895 Å and c=15.863 Å, which agree well (within 3%) with the experimental ones a=b=3.991 Å and c=16.333 Å.[9] Moreover, the itinerant FM of 2D vdW layered $Fe_{3+X}GeTe_2$ comes mainly from the intralayer FM couplings (the interlayer coupling being much weaker, see Table I) and is not much sensitive to the c-axis lattice parameter. Our test calculations show that when optimizing the c-axis lattice

parameter from the experimental c=16.333 Å to the theoretical c=15.672 Å (keeping a=b=3.991 Å unchanged), the calculated AFM1-FM energy differences change little from 470 meV/f.u. to 452 meV/f.u. Therefore, we keep using the experimental lattice constants but carry out atomic relaxation to study the magnetism mainly intralayer one) of $Fe_{3+X}GeTe_2$. In addition to LSDA, the LSDA plus Hubbard U (LSDA+U) method is employed[14] to include a moderate electron correlation of the constituent Fe atoms. We find that in the reasonable range of U=2-5 eV, the experimental ferromagnetic ground state of bulk Fe_3GeTe_2 is reproduced. Therefore, we have chosen the moderate U=3.5 eV (and Hund exchange J=0.9 eV) for the Fe 3d electrons, to calculate the magnetic properties.

In our LSDA calculations, the ferromagnetic (FM) state is much more stable than the nonmagnetic state (No-M) by 462 meV/f.u., as seen in Table S1. Therefore, Fe_3GeTe_2 has an intrinsic spin polarization, gaining Hund exchange energy at the magnetic Fe sites. The outer Fe1 and Fe2 atoms (nominal Fe^{3+}, see Figure 4b) have the spin moment of 1.99 μ_B, and the inner Fe3 atom (nominal Fe^{2+}) has 1.28 μ_B that is reduced by higher coordinations and stronger hybridizations. However, the inter-layer AFM state (with antiferromagnetically coupled FM layers) is calculated within LSDA to be more stable than the FM state by 21 meV/f.u., and this result is at odds with the experimental observation of the FM ground state. To cure this deficiency, we refer to a moderate electron correlation which appears quite often in the metallic transition-metal compounds. Then we have carried out LSDA+U (Coulomb repulsion) calculations to reproduce the FM ground state. As seen in Figure S17, the FM state gets more stable than the inter-layer AFM state with the tested U values of 2-5 eV, and therefore, we have chosen the mean value U=3.5 eV in the LSDA+U calculations for $Fe_{3+X}GeTe_2$.

Table S1| Relative total energy (meV/f.u.) and local spin moments (μ_B) of different magnetic states calculated by LSDA for bulk Fe_3GeTe_2

Magnetic state	ΔE	Fe1	Fe2	Fe3
FM	0	1.99	1.98	1.28
Inter-AFM	-21	2.20	2.20	1.18
No-M	462	--	--	--

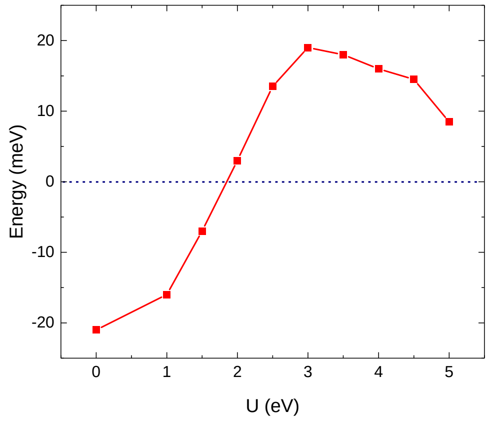

Figure S17. The energy of $E_{inter-AFM}$–E_{FM} versus U of Fe 3d electrons.

Note S6. Electrons doping in Fe_3GeTe_2

Through DFT calculations we find that starting from different positions of the extra Fe atom, in majority of the cases it ends up with the Fe atom residing in the van der Waals gap. The additional Fe atoms most probably lie in the interlayer interstitial region and provide extra carriers which could enhance the itinerant FM up to a particular concentration. To verify this, we have carried out additional calculations by adding extra electrons to the Fe_3GeTe_2 unit cell. Figure S18 shows that the energy difference between the FM and AFM1 increases up to the doping concentration of 1.6 e/fu. This similar trend with the calculated results in Figure 4a implies that the doped electrons play an important role in raising T_C of $Fe_{3+X}GeTe_2$ up to an optimal doping level.

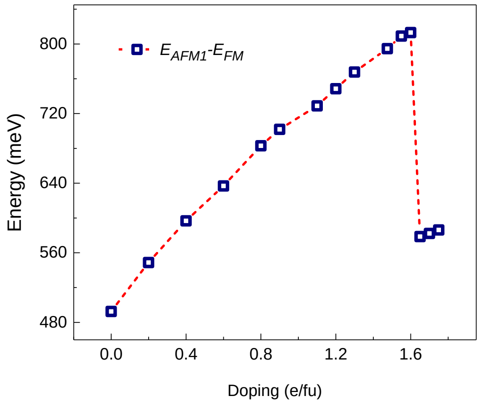

Figure S18. Total energy difference between the FM and AFM1 states calculated by LSDA+U as a function of additional electrons per fu.

References

1. Deng Y, Yu Y, Song Y *et al.* Gate-tunable room-temperature ferromagnetism in two-dimensional Fe_3GeTe_2. *Nature* 2018;**563**:94.

2. Qiu ZQ, Pearson J, Bader SD. Asymmetry of the spin reorientation transition in ultrathin Fe films and wedges grown on Ag(100). *Phys Rev Lett* 1993;**70**:1006–9.

3. Gradmann U. Magnetic Surface Anistropies. *J Magn Magn Mater* 1986;**54–57**:733–6.

4. Billas IML, Ch âtelain A, de Heer WA. Magnetism of Fe, Co and Ni clusters in molecular beams. *J Magn Magn Mater* 1997;**168**:64–84.

5. Stoner EC, Wohlfarth EP. A mechanism of magnetic hysteresis in heterogeneous alloys. *Phil Trans R Soc Lond A* 1948;**240**:599–642.

6. Verchenko VYu, Tsirlin AA, Sobolev AV *et al.* Ferromagnetic Order, Strong Magnetocrystalline Anisotropy, and Magnetocaloric Effect in the Layered Telluride $Fe_{3-\delta}GeTe_2$. *Inorg Chem* 2015;**54**:8598–607.

7. Tan C, Lee J, Jung S-G *et al.* Hard magnetic properties in nanoflake van der Waals Fe_3GeTe_2. *Nat Commun* 2018;**9**:1554.

8. May AF, Calder S, Cantoni C *et al.* Magnetic structure and phase stability of the van der Waals bonded ferromagnet $Fe_{3-x}GeTe_2$. *Phys Rev B* 2016;**93**:014411.

9. Deiseroth H-J, Aleksandrov K, Reiner C *et al.* Fe_3GeTe_2 and Ni_3GeTe_2 – Two New Layered Transition-Metal Compounds: Crystal Structures, HRTEM Investigations, and Magnetic and Electrical Properties. *Eur J Inorg Chem* 2006;**2006**:1561–7.
10. Tian C-K, Wang C, Ji W *et al.* Domain wall pinning and hard magnetic phase in Co-doped bulk single crystalline Fe_3GeTe_2. *Phys Rev B* 2019;**99**:184428.
11. Zhu J-X, Janoschek M, Chaves DS *et al.* Electronic correlation and magnetism in the ferromagnetic metal Fe_3GeTe_2. *Phys Rev B* 2016;**93**:144404.
12. Chen B, Yang J, Wang H *et al.* Magnetic Properties of Layered Itinerant Electron Ferromagnet Fe_3GeTe_2. *J Phys Soc Jpn* 2013;**82**:124711.
13. Zhuang HL, Kent PRC, Hennig RG. Strong anisotropy and magnetostriction in the two-dimensional Stoner ferromagnet Fe_3GeTe_2. *Phys Rev B* 2016;**93**:134407.
14. Anisimov VI, Aryasetiawan F, Lichtenstein AI. First-principles calculations of the electronic structure and spectra of strongly correlated systems: the LDA + U method. *J Phys Condens Matter* 1997;**9**:767.
